# Breeding Substrate Containing Distillation Residues of Mediterranean Medicinal Aromatic Plants Modulates the Effects of *Tenebrio molitor* as Fishmeal Substitute on Blood Signal Transduction and WBC Activation of Gilthead Seabream (*Sparus aurata*)

**DOI:** 10.3390/ani13152537

**Published:** 2023-08-06

**Authors:** Efthimia Antonopoulou, Markos Kolygas, Nikolas Panteli, Evangelia Gouva, Panagiota Kontogeorgiou, Konstantinos Feidantsis, Achilleas Chatzopoulos, Konstantina Bitchava, Christos Zacharis, Eleftherios Bonos, Ilias Giannenas, Ioannis Skoufos, Stefanos S. Andreadis, Georgios Skoulakis, Christos G. Athanassiou, Cosmas Nathanailides

**Affiliations:** 1 Department of Zoology, School of Biology, Aristotle University of Thessaloniki, 54124 Thessaloniki, Greece; 2 Department of Agriculture, University of Ioannina, 47100 Arta, Greece; kolygasmarkos@gmail.com (M.K.);; 3 Department of Fisheries and Aquaculture, University of Patras, 26504 Messolonghi, Greece; 4 Laboratory of Applied Hydrobiology, Department of Animal Science, School of Animal Biosciences, Agricultural University of Athens, 11855 Athens, Greece; 5 Skaloma Fishery [A.C], 46300 Sagaida, Greece; 6 School of Veterinary Medicine, Aristotle University of Thessaloniki, 54124 Thessaloniki, Greece; 7 Institute of Plant Breeding and Genetic Resources, Hellenic Agricultural Organization “DEMETER”, 57001 Thermi, Greece; 8 AgriScienceGEO, Melpomenis Str. P.O. Box 60561, Industrial Area of Thermi, 57001 Thessaloniki, Greece; 9 Department of Agriculture, Crop Production and Rural Environment, University of Thessaly, 38446 Nea Ionia, Greece

**Keywords:** *Tenebrio molitor*, gilthead sea bream, differential leukocyte count, MAPK signaling, heat shock proteins

## Abstract

**Simple Summary:**

Nowadays, aquaculture is the main source of fish for consumers, and its demand is constantly increasing. The diet of these fish requires protein for their growth, which is traditionally obtained from natural fish stocks. In recent years, efforts have been made to replace these feed ingredients with insect-derived meals, but it is necessary to investigate their impact on the growth and health status of the fish. In this study, two insect meals of *Tenebrio molitor* larvae reared in conventional substrates or substrates enriched with medicinal/aromatic plants (MAPs) were supplemented (10%) as fish meal replacements in the diets of gilthead seabream. After 12 weeks of experimental feeding, hematological and biochemical tests were conducted, revealing specific modulations in white blood cell and blood signal transduction patterns in the fish subjected to each experimental diet in relation to the new feed ingredients.

**Abstract:**

This work assesses the dietary use of two insect meals of *Tenebrio molitor* (TM) larvae reared in conventional (TM-10) or MAP-enriched substrates (MAP-TM-10) as fish meal replacements (10%) in the diets of gilthead seabream (*Sparus aurata*). Fish (*n* = 4500; 207.19 ± 1.47 g) were divided into three groups with triplicates: control (fed conventional diet), TM-10, and MAP-TM-10 groups. The fish were reared in floating cages for 12 weeks and the dietary effects on white blood cell activation, heat shock proteins, MAPKs, and apoptosis of the fish were evaluated. The MAP-TM-10 group exhibited the highest eosinophilic induction. Phosphorylated levels of p38 MAPK, p44/42 MAPK, HSP70, and HSP90 increased in the TM-10 and MAP-TM-10 groups. In terms of apoptosis, Bax levels were lower in the TM groups compared to the control, and the MAP-TM-10 group showed even lower levels than the TM-10 group. Bcl-2 levels increased in the TM-10 group compared to the control, and further increased in the MAP-TM-10 group. The Bax/Bcl-2 ratio, an apoptosis indicator, decreased in the TM groups, with the MAP-TM-10 group showing a further decrease compared to TM-10. These findings suggest that insects’ breeding substrate being enriched with MAPs modulated the effect of TM on cellular stress and apoptosis.

## 1. Introduction

With overfishing and declining fish stocks, alternative sources of dietary protein have become ever more important and of increasing interest in recent years, especially in aquaculture [1]. *Tenebrio molitor* (TM) (Coleoptera: Tenebrionidae), commonly known as yellow mealworm, is increasingly being used as a feed ingredient and substitute for fish meal in aquaculture [2]. On a proximal composition basis, TM is rich in crude proteins and lipids and low in ash and crude fibers [3], with an abundance of essential amino acids (EAAs) [4] and distinct fatty acids profile [5].

Several authors have conducted analyses on the impact of incorporating mealworms into livestock feeds to assess their potential and their effects on livestock growth, animal health, and meat quality. Numerous studies have indicated the efficacy of substituting fishmeal with mealworm meals in aquaculture production, particularly at inclusion rates of up to 25% [6]. Mealworm-based feeds have received positive evaluations for various freshwater species such as Nile tilapia (*Oreochromis niloticus*) [7,8,9], tench (*Tinca tinca*) [10], rainbow trout (*Oncorhynchus mykiss*) [11,12,13,14,15,16,17,18,19], yellow catfish (*Pelteobagrus fulvidraco*) [20], black bullhead catfish (*Ameiurus melas*) [21], European perch (*Perca fluviatilis*) [22,23,24], and African catfish (*Clarias gariepinus*) [25] and several marine water species such as gilthead sea bream (*Sparus aurata*) [1,11,12,13,26,27,28,29], European sea bass (*Dicentrarchus labrax*) [11,13,26], blackspot sea bream (*Pagellus bogaraveo*) [30], olive flounder (*Paralichthys olivaceus*) [31], red sea bream (*Pagrus major*) [32], Pacific white shrimp (*Litopenaeus vannamei*) [33], pearl gentian grouper (*Epinephelus lanceolatus*♂ *x Epinephelus fuscoguttatus*♀) [34], and black rockfish (*Sebastes schlegeli*) [35]. Despite the positive evaluations, it should be noted that high levels of inclusion, particularly in fish juveniles, could potentially result in performance decrease [3,35].

In recent years, the medicinal/aromatic plants (MAPs) market has been resilient in terms of growth and development in many Mediterranean countries [36], while India, Brazil, China, Indonesia, and the USA [37] are the top essential oil (EO)-producing countries [38]. The Mediterranean has been characterized as a biodiversity hotspot, with exceptional numbers of endemic plants. For instance, more than 7000 native plant taxa occur in the Greek region, with 22% being endemic [39]. The MAPs market focuses mainly on industrial applications [37]. The expanding demand for EOs and extracts of MAPs can be attributed to their diverse and extensive applications in various sectors such as the cosmetics, fragrance, and food flavoring industries and as industrial solvents [40].

EOs’ yield performances fluctuate among MAPs due to different harvest strategies, harvest intensities, and hydrological annual profiles [40]. Nonetheless, the post-distillation residual plant biomass results in a substantial number of wasted by-products due to its significant volume [41]. These by-products contain phenolic compounds [42] and, if used as feed additives, can have antioxidant and inhibitory effects upon fish and pathogens’ growth, respectively [11,43,44,45].

However, adjusting the composition of a diet can potentially result in nutrient deficiencies, particularly with formulated diets that rely on feedstuff processing techniques, which greatly influence the bioavailability of nutrients [46]. Such fish feed modifications can lead to diet-induced oxidative stress [26,47], thus affecting numerous cellular mechanisms. Heat shock proteins (HSPs), which, as molecular chaperones, assist in the conformational folding and translocation of proteins [48,49], are implicated in the immediate response to exposure to a multitude of stressors [50]. External stimuli including dietary-related factors [47,51] may also modulate the activation of mitogen-activated protein kinases (MAPKs), which transduce signals for the regulation of several cellular processes such as development and apoptosis [52,53]. The latter is crucial under the stress regime for the homeostatic removal of damaged or superfluous cells [54].

This paper aims to evaluate the impact of the use of *T. molitor* larvae as a partial fish meal substitute and, at the same time, examine if the enrichment of the substrate of the larvae with post-distillation MAP residues can benefit and differentiate the produced insect meal from conventional meals by examining different stress and inflammatory biomarkers in the blood of gilthead seabream fed these meals. Specifically, peripheral white blood cell activation, heat shock proteins, mitogen-activated protein kinases, and apoptotic machinery were assessed.

## 2. Materials and Methods

### 2.1. Rearing of Tenebrio Molitor and Production of Insect Meals

Το produce the insect meals examined in this trial, *T. molitor* larvae were reared in two different substrates. The first meal (“conventional”) was created from larvae reared on a conventional substrate, while the second meal (“enriched”) was created from insects reared on a substrate partially enriched (20%) with plant material from processed residues from the distillation of the following medicinal aromatic plants: Greek oregano *(Origanum vulgare* subsp. *hirtum*), thymus (*Thymus vulgaris*), sage (*Salvia officinalis*), rosemary (*Rosmarinus officinalis*), and their essential oils and linseed (*Linum usitatissimum*), sea fennel (*Crithmum maritimum*), and olive (*Olea europaea* subsp. *europaea*). All the above were added in analogous percentages corresponding to the 20% of the insect diet (approx. 2.9% each). Insects were reared for a period of four months in total, starting from newly hatched larvae until the stage of late-instar larvae, i.e., prior to pupation, as suggested by Rumbos et al. [55], which was the instar that was used in the feeding trials. After collection, the insect larvae were air-dried for 48 h at a temperature of 38 °C using a VK981RH oven (Vencon-N.Varsos A.E., Athens, Greece). The dried material was kept frozen (−20 °C) until it was used for the preparation of the fish diets.In the conventional and enriched rearing substrates, the protein content of the insect meal was 56.25% and 55.16%, respectively. The total phenolics (g GAE/g DW) content of the insect meal in the conventional and enriched insect rearing substrates was 314.10% and 257.26%, respectively.

### 2.2. Fish Diets

Three different diets were prepared as described in Table 1: (a) a diet for the control group that was prepared with fishmeal as the main protein source (hereinafter control diet); (b) a diet with partial substitution of fishmeal with 10% of the “conventional” insect meal (hereinafter TM-10); (c) a diet with partial substitution of fishmeal with the “enriched” insect meal (hereinafter MAP-TM-10).The diet for the control group was prepared by mixing a commercial feed (Praxis Fish Feeds, Piraeus, Greece) appropriate for gilthead sea bream (*Sparus aurata*) with fish meal and wheat (extruded). The diets for the other two groups were prepared by combining the same commercial feed with either the “conventional” (TM-10) or the “enriched” (MAP-TM-10) dried insect meals.

**Figure 1:**
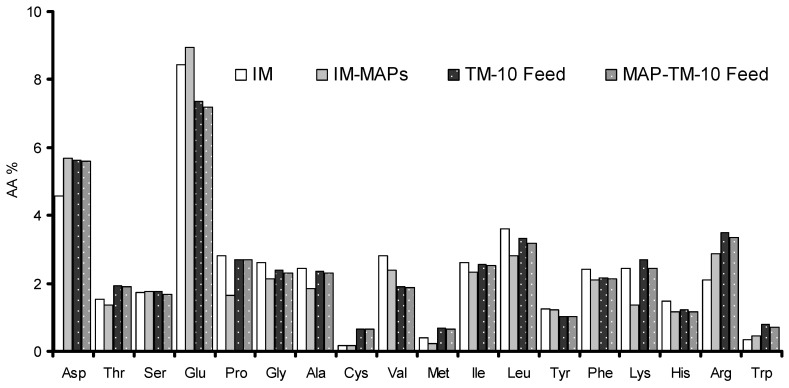
Amino acid (AA%, dry weight basis) concentrations in TM meal produced in conventional (IM, dotted white bars) or in enriched substrate (IM-MAPs, grey bars) and in fish feed of the TM-10 (dotted black bars) and the MAP-TM-10 group (dotted grey bars). Aspartic acid—Asp, threonine—Thr, serine—Ser, glutamic acid—Glu, proline—Pro, glycine—Gly, alanine—Ala, cysteine—Cys, valine—Val, methionine—Met, isoleucine—Ile, leucine—Leu, tyrosine—Tyr, phenylalanine—Phe, lysine—Lys, histidine—His, arginine—Arg, tryptophan—Trp.

For the preparation of each diet, the feed ingredients were initially ground in a hammer mill, then mixed, pelleted (5 mm) using a pellet mill, air-dried (25 °C), and stored. The three diets were formulated to be isonitrogenous and isocaloric and to meet the nutrient requirements of the gilthead seabream. The AA content of each feed was analyzed in a near-infrared reflectance spectrometer (Figure 1) (DA 7250 NIR Perten analyzer, Perkin Elmer)and the crude protein content of the insect meals was analyzed according to the method 968.06 of the Association of Official Analytical Chemists (AOAC) [24] for crude protein [56]. The total phenolic content of the diets was analyzed with the Folin–Ciocalteu method as described by Vasilopoulos et al. [57].

### 2.3. Dietary Experiments, Rearing Facilities, and Experimental Conditions

A Growth trial (three independent replicates) was performed in an aquaculture facility in the northwest region of Greece (Skaloma S.A). A total of 4500 (207.19 ± 1.47 g average initial body weight) gilthead sea bream (*Sparus aurata*) of both sexes were individually weighed and were randomly allocated into 9 floating cages (500 individuals per cage). A total of 3 triplicate experimental groups were formed: the control group A, which was fed the control diet; the experimental group B, which was fed with TM-10; the experimental group C, fed with MAP-TM-10. Each diet was administered twice a day (09:00 h and 16:00 h) at a 1.5 feeding rate (FR), 7 days per week, for 4 consecutive weeks. All fish were considered clinically healthy based on an external examination for any signs of infestation, skeletal deformities, soft tissue deformities, ocular injuries, or wounds. Prior to the experiment, anti-cohabitation measures took place to ensure equal growth and feed intake potential for all experimental fish. Throughout the duration of the trial, all experimental groups were subjected to similar natural environmental conditions (photoperiod, water temperature, water depth, and cohabitation potential). The exact amount of feed distributed to each sea cage (feed intake) was recorded daily. Feeds were administered over the whole water surface to be simultaneously accessible to all the fish. During the trial, all groups were inspected daily to check mortality.

### 2.4. Sampling Procedures

Fish were randomly sampled from all groups before feeding through confinement and netting in the tanks. Fish were fasted for 18 h before sampling to minimize handling stress. The fish were anaesthetized prior to blood sampling using prescribed buffered Benzocaine at a concentration of 400 ppm. Following the anaesthetization procedure, the fish were individually weighed to the nearest 0.1 g (Kern, PES 2200-2M), their fork length (L) was recorded, and, thereafter, they underwent venipuncture.

### 2.5. Manual Hematological Analysis

**Figure 2:**
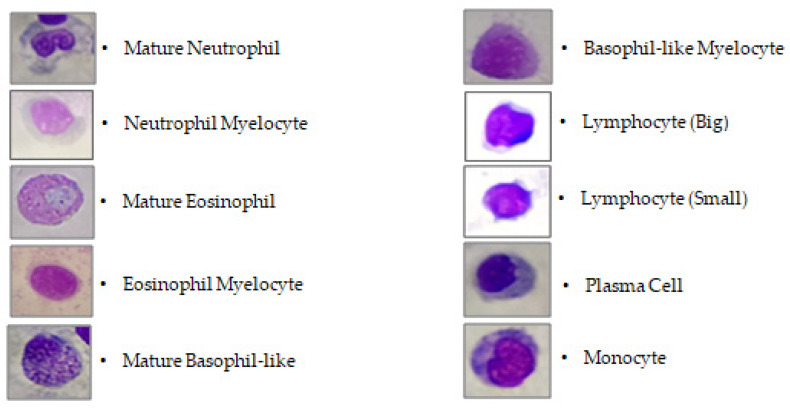
Morphology of mature and blastic leukocyte cells in gilthead sea bream’s peripheral blood.

At the end of the 4-week experiment, 5 gilthead sea bream were randomly selected from each of the 9 floating cages (45 fish—15 per group). Blood samples were obtained from the caudal vein of every fish using a 20 G × 1½ syringe without any anticoagulant agent to assess the differential leukocyte count (DLC) [58]. Blood smears were taken immediately given the rapid blood clotting of gilthead sea bream. A blood drop (without anticoagulants) was applied on a degreased glass slide and was smeared out, dried at room temperature, fixed with methanol, and stained (Hemacolor^®^ Rapid Staining Set (Merck KGaG, Darmstadt, Germany). Each smear was divided into 6 randomly selected areas of 1 mm^2^, and the absolute percentage of each type of white blood cell was counted (lymphocytes, neutrophils, eosinophils, monocytes, and their blast forms were calculated as a percentage). Smears were studied using an Olympus CX23 light microscope. The cellular size, nuclear morphology, and cytoplasmic staining patterns were the criteria for the cells’ identification and determination [59,60,61]. The DLC was recorded as a percentage of a particular cell type [58]. Mature and blastic cells were involved in the leukogram (Figure 2), but thrombocytes and thrombocyte-like cells were excluded in the white blood cell parameters, since they represent a distinct blood cell type [62]. A total of 270 (45 fish smears × 6 randomly selected divisions/smear) blood assays were conducted.

### 2.6. Determination of HSPs, MAPKs, and Apoptosis

The preparation of whole blood samples and the immunoblot analysis were conducted following well-established protocols. In this study, for SDS-PAGE, equivalent amounts of proteins from whole blood were separated using acrylamide and bisacrylamide gels with concentrations of either 10 or 0.275% (*w*/*v*). Subsequently, the proteins were transferred electrophoretically onto nitrocellulose membranes. The antibodies used included polyclonal rabbit anti-heat shock protein 70 kDa, polyclonal rabbit anti-heat shock protein 90 kDa, polyclonal rabbit anti-phospho-p38 MAPK (Thr180-Tyr182) (9211, Cell Signaling), monoclonal rabbit anti-phospho p44/42 MAPK (4376, Cell Signaling), polyclonal rabbit anti-bcl2 (7973, Abcam), polyclonal rabbit anti-bax (B-9) (7480, Santa Cruz Biotechnology, Dallas, TX, USA), and actin (anti-β actin 3700, Cell Signaling, Beverly, MA, USA). To ensure quality transfer and protein loading, Ponceau stain was utilized for Western blotting. Bands were detected using enhanced chemiluminescence, and quantification was performed using laser scanning densitometry with (GelPro Analyzer Software 32, GraphPad, San Diego, CA, USA).

### 2.7. Statistical Analysis and Formulae

Three independent replicates were used in all analyses, with the exception of differential blood count, where six independent replicates were used. Data are presented as the mean ± standard error of mean (SEM). Mean values were compared among treatments using one-way analysis of variance (ANOVA). Post hoc comparisons were analyzed using Tukey’s HSD, the Bonferroni and Holms test, and Scheffe’s multiple comparison test. The differences were determined to be significant at *p* < 0.05. All statistical analyses were carried out using SPSS version 21.0 (SPSS Inc., Chicago, IL, USA). All formulae used are presented below:

Weight gain (WG, %) = [(final body weight − initial body weight)/initial body weight] × 100;

Specific growth rate (SGR, %/day) = [(ln final body weight − ln initial body weight)/days of experiment] × 100;Feed conversion ratio (FCR) = feed intake/wet weight gain;

Protein efficiency ratio (PER) = weight gain/protein intake;

Survival (%) = (final number of fish/initial number of fish) × 100;

Differential leukocyte count (%) = % of a given white cell type = 100% × absolute number of a given white cell type (cells/μL)/total WBCs (cells/μL).

### 2.8. Experiment—Ethics Aspects

The insects used in this study originated from a stock colony maintained at the Entomology Lab of the Institute of Plant Breeding and Genetic Resources (IPGRB) of the Hellenic Agricultural Organization Demeter in Thermi, Greece. The insects were kept under standard conditions: temperature 26 °C; 60% relative humidity; 8:16 h (L:D) photoperiod.

The experimental protocol for this trial was reviewed and approved by the Research Ethics Committee of the University of Ioannina (Greece) (protocol No 56652, 26 November 2021). The trial involving gilthead sea bream was conducted by accredited scientists at an aquaculture facility in the northwest region of Greece (Skaloma S.A). The accredited scientists were responsible for the supervision of animal care, procedures, sampling, anesthesia, and euthanasia of experimental fish. The experimental procedures followed the guidelines outlined in the current European Directive (2010/63/EU) for the protection of animals used in scientific research. The use of dried *T. molitor* as food for humans and animals is considered safe under EU Regulation 882/2021, and any by-products obtained from vertebrate experimental animals were handled in accordance with Regulation (EC) No 1069/2009.

## 3. Results

### 3.1. Growth Performance and Relevant Growth Indices

There was no significant difference in the mean weight gain among the different groups (*p* > 0.05). The specific growth rate (SGR), feed conversion ratio (FCR), and survival rate also displayed no significant differences (*p* > 0.05) among the treatments (Table 2).

### 3.2. Differential Leukocyte Count (DLC)

**Figure 3:**
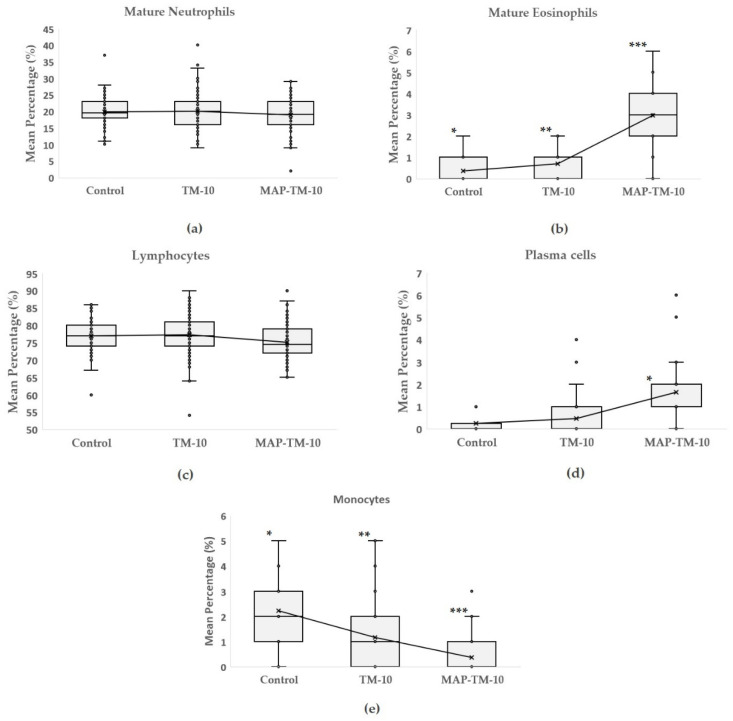
Box-and-whisker plots of mean percentage of mature neutrophils (**a**), mature eosinophils (**b**), lymphocytes (**c**), plasma cells (**d**), and monocytes (**e**) of the three experimental groups. Significant differences among groups are denoted with asterisks (* *p* < 0.05; ** *p* < 0.01; *** *p* < 0.001). The empty circles in the boxplots denote values being identified as outliers.

The results in Figure 2 show the proportions of mature neutrophils, mature eosinophils, lymphocytes, plasma cells, and monocytes of the three groups. Among the three groups, no significant differences (*p* > 0.05) were observed between the mean percentage values of mature neutrophils and lymphocytes. However, a significant statistical difference was observed between the groups in terms of mature eosinophils, with the MAP-TM-10 group showing the highest eosinophilic induction (*p* < 0.01), followed by the TM-10 group (*p* < 0.05). A similar pattern was observed for plasma cells, with the MAP-TM-10 group exhibiting the highest mean percentage values and a significant statistical difference from the TM-10 and control groups (*p* < 0.01). Monocytes demonstrated significant statistical differences among the three groups (*p* < 0.01). The lowest mean percentage value was observed in the MAP-TM-10 group, while the second highest was observed in the TM-10 group, (Figure 3).

### 3.3. Heat Shock Induction

**Figure 4:**
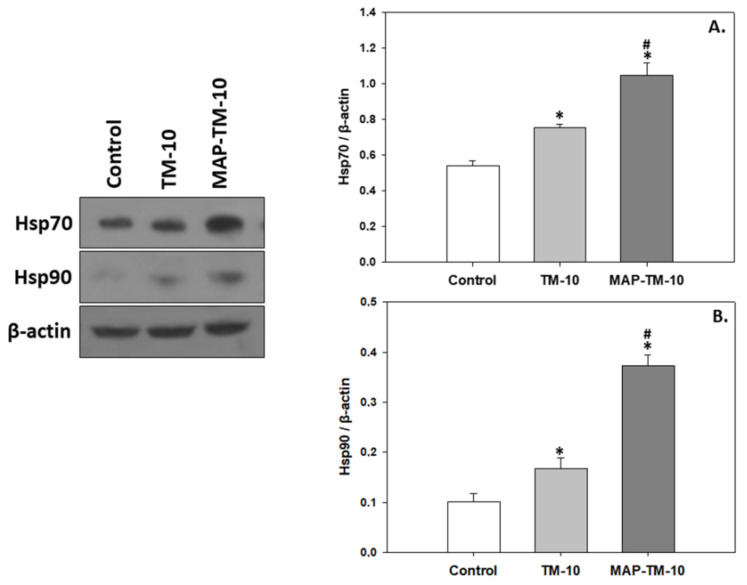
Levels of Hsp70 (**A**) and Hsp90 (**B**) (mean ± SEM) in the whole blood of *S. aurata* under the control, TM-10, and MAP-TM-10 feeding regimes. The quantitative histograms show the changes in the abovementioned indicators, normalized with β-actin. Representative blots are shown. *n* = 5 preparations from different animals. Significant differences (*p* < 0.05) compared to the control are presented as *, while significant differences (*p* < 0.05) between TM-10 and MAP-TM-10 are presented as #.

Both HSP members examined in the present study exhibited an increasing pattern in both insect-based feeding regimes compared to the control group. Specifically, the TM-10 group showed statistically significantly increased levels (*p* < 0.05) of both HSP70 and HSP90 compared to the control. Moreover, the MAP-TM-10 group exhibited significantly (*p* < 0.05) increased levels of HSP70 (*p* < 0.05) and HSP90 (*p* < 0.001) in the fish whole blood compared to the control(Figure 4A,B).

### 3.4. MAPK Signaling

**Figure 5:**
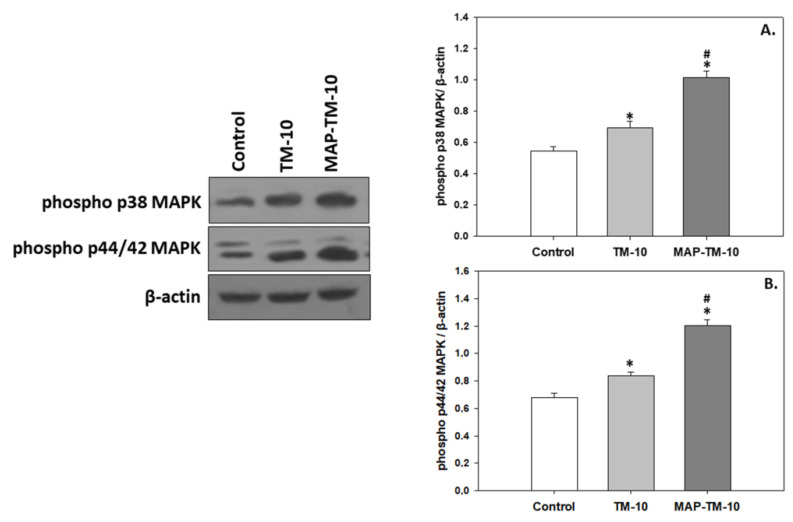
Phosphorylated levels of p38 MAPK (**A**) and p44/42 MAPK (**B**) (mean ± SEM) in the whole blood of *Sparus aurata* under the control, TM-10, and MAP-TM-10 feeding regimes. The quantitative histograms show the changes in the abovementioned indicators, normalized with β-actin. Representative blots are shown. *n* = 5 preparations from different animals. Significant differences (*p* < 0.05) compared to the control are presented as *, while significant differences (*p* < 0.05) between TM-10 and MAP-TM-10 are presented as #.

The MAPK phosphorylation exhibited a similar pattern to the HSP induction. Specifically, the TM-10 feeding regime showed significantly increased (*p* < 0.05) phosphorylated levels of p38 MAPK and p44/42 MAPK compared to the levels exhibited in the control. Additionally, the MAP-TM-10 feeding regime showed significantly increased (*p* < 0.05) phosphorylated levels of both p38 MAPK and p44/42 MAPK in the fish whole blood compared to the control (Figure 5A,B).

### 3.5. Apoptosis

**Figure 6:**
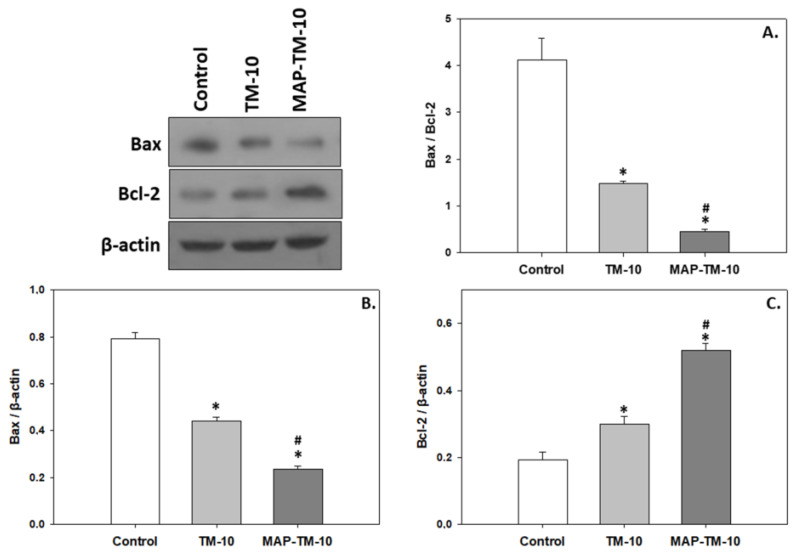
Levels of Bax/Bcl-2 ratio (**A**), Bax (**B**), and Bcl-2 (**C**) (mean ± SEM) in the whole blood of *Sparus aurata* under the control, TM-10, and MAP-TM-10 feeding regimes. The quantitative histograms show the changes in the abovementioned indicators, normalized with β-actin. Representative blots are shown. *n* = 5 preparations from different animals. Significant differences (*p* < 0.05) compared to the control are presented as *, while significant differences (*p* < 0.05) between TM-10 and MAP-TM-10 are presented as #.

The Bax and Bcl-2 proteins, which promote and prevent apoptosis, respectively, indicate dietary-induced effect. In contrast to the pattern observed in both HSPs and MAPKs, Bax levels exhibited statistically significant decreased levels (*p* < 0.05) in whole blood of fish fed the TM-10 regime compared to the control. The MAP-TM-10 regime showed even more decreased levels (*p* < 0.05) of Bax compared to the TM-10 regime (Figure 6B). However, Bcl-2 exhibited an opposite pattern, with TM-10 resulting in increased levels compared to the control(*p* < 0.05) and MAP-TM-10 in even more increased levels (*p* < 0.05) compared to the control and also the TM-10 feeding regime (Figure 6C). As expected from the above, the Bax/Bcl-2 ratio, which is a potent apoptotic indicator, depicted statistically significant decreased levels (*p* < 0.05) in the whole blood of fish fed with both the TM-10 and MAP-TM-10 regimes. Moreover, the MAP-TM-10 regime exhibited a statistically significant decrease (*p* < 0.05) compared to the TM-10 feeding regime (Figure 6A).

## 4. Discussion

Initially, publications regarding the use of TM larvae in gilthead seabream primarily focused on growth performance, feed convertibility, and fillet composition, among other indices [29]. However, recent studies began to investigate the molecular, biochemical, and physio-pathological pathways triggered in fish by supplementation of fish meal with insect meal [13,26].

### 4.1. Gilthead Sea Bream Growth Performance

As stated in previous studies, in the context of growth performance, the replacement of 25% fishmeal (FM) with TM larvae meals has shown encouraging results in terms of growth indicators, although a slight decrease in protein efficiency ratio (PER) and feed conversion ratio (FCR) was noticed compared to the FM-supplemented group [28]. In some cases, higher replacement percentages (50% FM) resulted in improved gut microbial diversity [11] and amino acid composition [2,12], while in some others, they resulted in increased operational taxonomic units without corresponding to higher diversity in gut microbiota [1]. Our results are in accordance with previous literature findings from studies that examined similar insect meal supplementation rates in terms of growth performance indices of other farmed fish species [3,13,16,17,18,19,20,21,22] and gilthead seabream. [63]. More specifically, both the TM-10 insect meal supplementation and MAP-TM-10 groups had no adverse effects on WG, SGR, and FCR in comparison to the control group. The results show that there were no significant differences in PER between the control group (A), the TM-10 group (B), and the MAP-TM-10 group (C). This means that all three groups were able to retain protein equally well, which was reflected in the PER (Table 2). In fact, the addition of TM-10 or MAP-TM-10 to the diet did not have a significant effect on protein retention and the values were within the range of PER reported by Piccolo et al. [38] in their study on the effects of *T. molitor* larvae meal on the growth performance of gilthead seabream.

### 4.2. White Blood Cell Modultation

Blood analysis in fish has become an essential method for assessing stress and inflammatory biomarkers. Unlike the liver, where these biomarkers are often rapidly cleared, blood allows for easy detection and measurement of such biomarkers, providing valuable insights into the physiological responses of fish to different stressors and environmental challenges [63]. Blood tests on fish have been conducted for several decades, both in laboratory and field settings, to evaluate endocrine, reproductive, and immune functions, as well as maturation, nutrition, and health status [64]. The primary stress responses involve the release of catecholamines and corticosteroids [64]. Subsequently, secondary stress responses occur and have various effects on multiple tissues, including the blood. These secondary responses can include accelerated energy mobilization through glucose, changes in hydromineral balance, and increased lactate levels, as well as decreased blood pH, hematocrit, and leukocyte activation [65,66,67].

One interesting finding was the significant increase in eosinophil values among the groups, with higher values observed in the MAP-TM-10 group. At first glance, the results may seem contradictory, as there are several reports on the immunoregulatory capacity of essential oils in humans [68,69,70,71], though increased eosinophil values have been observed in Nile tilapia after administration of oregano essential oil (although without statistically significant differences compared to the controls) [72]. On the other hand, one could hypothesize that the observed eosinophilia is a reactive response to the insect meal substitute. Henry et al. [27] reported no significant changes in terms of gilthead seabream lymphocytes and eosinophils between their control and 10% full-fat substitute group (i.e., *Zophobas morio* (F.) larvae) after 100 days of administration. It is important to consider the heterogeneity of the seven different MAP residues used in our study, which, in combination with TM, may have resulted in this eosinophilic induction.

Another important finding is the significant decrease in monocyte cells (TM-10 and MAP-TM-10 groups) compared to the control, with the lowest percentage values observed in the MAP-TM-10 group. Similar monocyte pattern findings were reported by Henry et al. [27] following insect meal inclusion. Moreover, it is worth noting that MAP residues have been associated with potential antibacterial properties due to the presence of phenolic compounds [43,73]. In the present study, the antibacterial activity of MAP residues may have contributed to the significant reduction in monocytes observed in the MAP-TM-10 group, amplifying the effect. Like other vertebrates [74], the relative ratios of specific leukocyte populations in fish blood can provide valuable information about their response to specific treatments or environmental factors. Changes in the number of mature neutrophils and leukocytes and the ratio of neutrophilic/heterophilic granulocytes can indicate stress-induced WBC activation [67,75,76,77]. However, in the present work, there were no apparent differences among the three experimental groups in the number of mature neutrophils and leukocytes. This suggests that the dietary treatment did not induce any changes in WBC activation in the experimental fish, suggesting a well-tolerated immune homeostatic capacity.

### 4.3. Apoptosis, Heat Shock Proteins, and MAPK Signaling

Recent studies have investigated the potential impact of TM on the pathophysiology (physiological dysfunction) of gilthead seabream. For instance, Mente et al. [13] reported that dietary inclusion of 25% TM larvae meal in farmed fish led the induction of TM-related oxidative stress, which activated cell death mechanisms in the liver.

In this context, Bousdras et al. [26] exemplified that apoptosis was induced in seabream’s heart following the inclusion of 25% TM. However, tissue specificity was apparent in the abovementioned study, considering that apoptosis was suppressed in the muscle [26], which is in accordance with the present findings for the blood of both experimental diet groups. Numerous studies have highlighted that the impact of dietary insect meal highly depends on the levels of inclusion [78,79,80]. The substitution of fishmeal with mealworm meal at 75% provoked hepatic apoptosis in largemouth bass (*Micropterus salmoides*) compared to lower inclusion levels, which was attributed to the decreased feed utilization due to intestinal damage under that feeding regime [80]. Considering that growth performance indices displayed no alteration among the three diets herein, it is likely that such a low level of substitution exerts no detrimental effects that may induce apoptotic cell death. In addition, the enrichment of *T. molitor* substrate with MAP seems to strengthen the anti-apoptotic effects. Plant compounds have been shown both in vivo and in vitro to up-regulate Bcl-2 and suppress pro-apoptotic cytochrome c release from mitochondria in rats [81,82]. Furthermore, the ameliorative effects of herbal extracts in terms of apoptosis have also been observed in the breast tissue of broiler chicken following dietary supplementation [83].

MAPs are known to have antioxidant properties, and they have been shown to enhance the total phenolic and flavonoid content as well as the antioxidant potential of *T. molitor* [41]. Likewise, in the present study, the MAP-TM-10 group had a significantly higher level of total phenolics than the control group, which may have contributed to the anti-apoptotic effects observed in the MAP-TM-10 group.

Contrary to the apoptosis suppression, both insect-based diets led to the induction of HSPs and the activation of MAPKs compared to the control. In agreement with the present findings, several fish species, including gilthead seabream, exhibited up-regulation of HSPs in response to dietary inclusion of insect meal [26,84,85]. However, the induced HSP expression in the abovementioned studies was observed under considerably higher levels of inclusion compared to the 10% fishmeal substitution of the present study. Therefore, it is likely that the apparent HSP increase may lean more towards the recruitment of molecular chaperones for the protein metabolism rather than the involvement in nutrient stress response. Insect meals have been previously demonstrated to modify the amino acid catabolism in fish species such as rainbow trout, tench, and gilthead seabream [10,86]. Specifically, the elevated free amino acids and peptides from insect-based diets may favor protein synthesis [85], which requires HSPs for the proper folding of newly synthesized polypeptides [72]. Furthermore, the higher HSP levels in the gilthead seabream fed the MAP-enriched insect meal may also imply involvement in protein metabolism, considering that previous studies have demonstrated that supplementation of plant organic compounds contributes to the increase in free amino acids [87,88]. Interestingly, Lee et al. [89] indicated that garlic extract supplementation in sterlet sturgeon (*Acipenser ruthenus*) was accompanied by elevated circulating insulin, which may enhance the blood transfer of free amino acid to muscle. In addition, the regulation of HSP induction in fish has been proposed to partially involve the activation of MAPKs [90], which may explain the identical expression pattern shared among the two protein families herein. The tendency for a similar modification pattern of HSPs and MAPKs was previously reported by Stenberg et al. [91] in Atlantic salmon following dietary substitution of fish meal with black soldier fly larvae meal. However, further research is required, especially for insect meal-induced alterations in the activation of MAPKs. Seo et al. [92] suggested that ethanol extracts of *T. molitor* larvae suppress adipogenic differentiation via p44/42 MAPK inactivation and p38 MAPK activation in mice.

## 5. Conclusions

The results of this study suggest that the addition of TM-10 or MAP-TM-10 insect meal to the diet of gilthead sea bream does not have a significant effect on SGR, PER, or FCR. However, there were some differences observed in the white blood cell profiles of the fish in the different groups. This suggests that the MAP-TM-10 diet may have a different effect on the immune system of the fish than the other two diets. Plant extracts have been found to promote various activities in fish and shrimp aquaculture, including anti-stress effects, growth promotion, appetite stimulation, enhancement of tonicity, immune stimulation, maturation of culture species, and antibacterial properties. MAPs (medicinal aromatic plants) were not directly added to the diets in this experiment, but rather served as the substrate for *T. molitor* larvae. As a result, the only way MAPs might have impacted the fish was through their consumption of insect meal produced in MAP-enriched substrate. Given the tissue-specific nature of fish biochemical processes [93,94,95], as well as the growing need for sustainable aquaculture practices [96], more research is needed on the molecular and metabolic pathways in insects reared in MAP-enriched substrates. Additionally, future research should also look into whether MAP-enriched insect meal has an effect on the fatty acid profiles of the fish flesh. This would help to elucidate the mechanism implicated in the modulation of physiological parameters and the potential effect on fish flesh quality of fish fed with MAP-TM-10 feed.

## Figures and Tables

**Table 1:** Composition of the experimental diets.

Ingredients (g/kg)	Control	TM-10	MAP-TM-10
Fish feed	900	900	900
Wheat (extruded)	60	0	0
Fish meal (71% CP)	40	0	0
“Conventional” insect meal (35% CP)	0	100	0
“Enriched” insect meal (35% CP)	0	0	100
**Calculated analysis** (**as fed**)			
Digestible energy (Mj/kg)	15.79	15.80	15.80
Total phenolic content (TPC) of fish feed (mg GAE/L extract phenol)	12.42	44.13	56.08
Crude protein (g/kg)	439.9	440.0	440.0
Nitrogen-free extract (g/kg)	163.8	126.0	126.0

**Table 2:** Growth performance indices between dietary groups.

Parameters	Control (A)	TM-10 (B)	MAP-TM-10 (C)	Significance
Initial weight (g)	208.84 ± 2.88	208.19 ± 7.04	205.88 ± 8.81	Ns ^1^
Final weight (g)	334.92 ± 7.77	340.02 ± 3.72	343.90 ± 5.68	Ns
Weight gain (g)	129.08 ± 7.50	131.83 ± 4.09	138.02 ± 3.16	Ns
^2^ FR (%)	1.5	1.5	1.5	Ns
^3^ SGR (%/day)	0.58 ± 0.03	0.61 ± 0.03	0.63 ± 0.03	Ns
^4^ FCR (%)	1.19 ± 0.01	1.14 ± 0.03	1.08 ± 0.02	Ns
^5^ PER	2.04 ± 0.11	1.99 ± 0.06	2.10 ± 0.05	Ns
Survival rate (%)	98.08	99.23	98.46	Ns

^1^ non-significant statistical difference among groups (α = 0.05). ^2^ FR = feeding rate; ^3^ SGR = specific growth rate; ^4^ FCR = feed conversion ratio; ^5^ PER = protein efficiency ratio.

## Data Availability

The data presented in this study are available upon request from the corresponding author.
